# LINE-1 Retroelements Complexed and Inhibited by Activation Induced Cytidine Deaminase

**DOI:** 10.1371/journal.pone.0049358

**Published:** 2012-11-02

**Authors:** Mirjam Metzner, Hans-Martin Jäck, Matthias Wabl

**Affiliations:** 1 Department of Microbiology and Immunology, University of California San Francisco, San Francisco, California, United States of America; 2 Division of Molecular Immunology, Department of Internal Medicine III, Nikolaus-Fiebiger-Center, University of Erlangen-Nürnberg, Erlangen, Germany; Institut Jacques Monod, France

## Abstract

LINE-1 (abbreviated L1) is a major class of retroelements in humans and mice. If unrestricted, retroelements accumulate in the cytoplasm and insert their DNA into the host genome, with the potential to cause autoimmune disease and cancer. Retroviruses and other retroelements are inhibited by proteins of the APOBEC family, of which activation-induced cytidine deaminase (AID) is a member. Although AID is mainly known for being a DNA mutator shaping the antibody repertoire in B lymphocytes, we found that AID also restricts de novo L1 integrations in B- and non-B-cell lines. It does so by decreasing the protein level of open reading frame 1 (ORF1) of both exogenous and endogenous L1. In activated B lymphocytes, AID deficiency increased L1 mRNA 1.6-fold and murine leukemia virus (MLV) mRNA 2.7-fold. In cell lines and activated B lymphocytes, AID forms cytoplasmic high-molecular-mass complexes with L1 mRNA, which may contribute to L1 restriction. Because AID-deficient activated B lymphocytes do not express ORF1 protein, we suggest that ORF1 protein expression is inhibited by additional restriction factors in these cells. The greater increase in MLV compared to L1 mRNA in AID-deficient activated B lymphocytes may indicate less strict surveillance of retrovirus.

## Introduction

Activation-induced cytidine deaminase (AID) is a mutator in B lymphocytes that deaminates cytosine to uracil in DNA [1]. The classic function of AID is to mediate somatic hypermutation and class switch recombination of immunoglobulin (Ig) genes in antigen-stimulated B cells – processes important for the generation of highly specific antibodies with various effector functions [2]. But this function is not nearly as exclusive as previously thought. In addition to the Ig locus, AID also mutates other loci throughout the genome [3], [4]. Moreover, AID is thought to be critical in epigenetic reprogramming and potentially in restricting the inheritance of epimutations in mammals. Genome-wide erasure of DNA methylation in mouse primordial germ cells is affected by AID deficiency [5], and AID is required for DNA demethylation and initiation of nuclear reprogramming toward pluripotency in human somatic cells [6].

AID is the founding member of the APOBEC family of cytidine deaminases [7], [8]. Most of the APOBEC proteins mediate innate immunity by restricting retroviruses and other retroelements, including long interspersed nuclear element-1 (LINE-1, L1) [9], [10]. Retroelements are mobile segments of DNA that make up about 40% of the mammalian genome [11]. L1 elements make up 17–20% of the human and mouse genomes [12]. Of the approximately half a million L1 copies in humans and mice, most are truncated and inactive, but 100 human and 3,000 mouse L1 sequences are full-length and transposition competent [12]. If unrestricted, L1 and other retroelements are transcribed; their RNA, cDNA and protein accumulates in the cell; and their cDNA inserts into the host genome, with the potential to cause diseases ranging from cancer to autoimmunity [11], [13]–[16]. Until recently, it was believed that L1 elements were expressed only in embryonic cells, germ cells and cancer cells [11]. But active L1 elements have also been found in somatic cells [17]–[19].

APOBEC3G (abbreviated A3G) suppresses Alu retroelements by sequestering their RNA into large complexes in the cytoplasm, away from the nucleus, where they insert into the genomic DNA [20], [21]. The mechanism of inhibition of L1 retrotransposition by A3 proteins, however, is unknown. Recent data suggest that AID also functions in innate immunity: (i) AID protects pre-B cells against transformation by the oncogenic Abelson virus [22], and (ii) in non-B-cell lines, ectopic AID can restrict endogenous L1 and MusD retroelements [23]. In the current study, we investigated whether AID also functions as an inhibitor of retroelements, similar to other members of the APOBEC family. Our data show that AID does restrict L1 retroelements, apparently by forming large cytoplasmic complexes with their mRNA, binding to and decreasing the steady-state level of proteins encoded by them, and inhibiting their retrotransposition.

**Figure 1:**
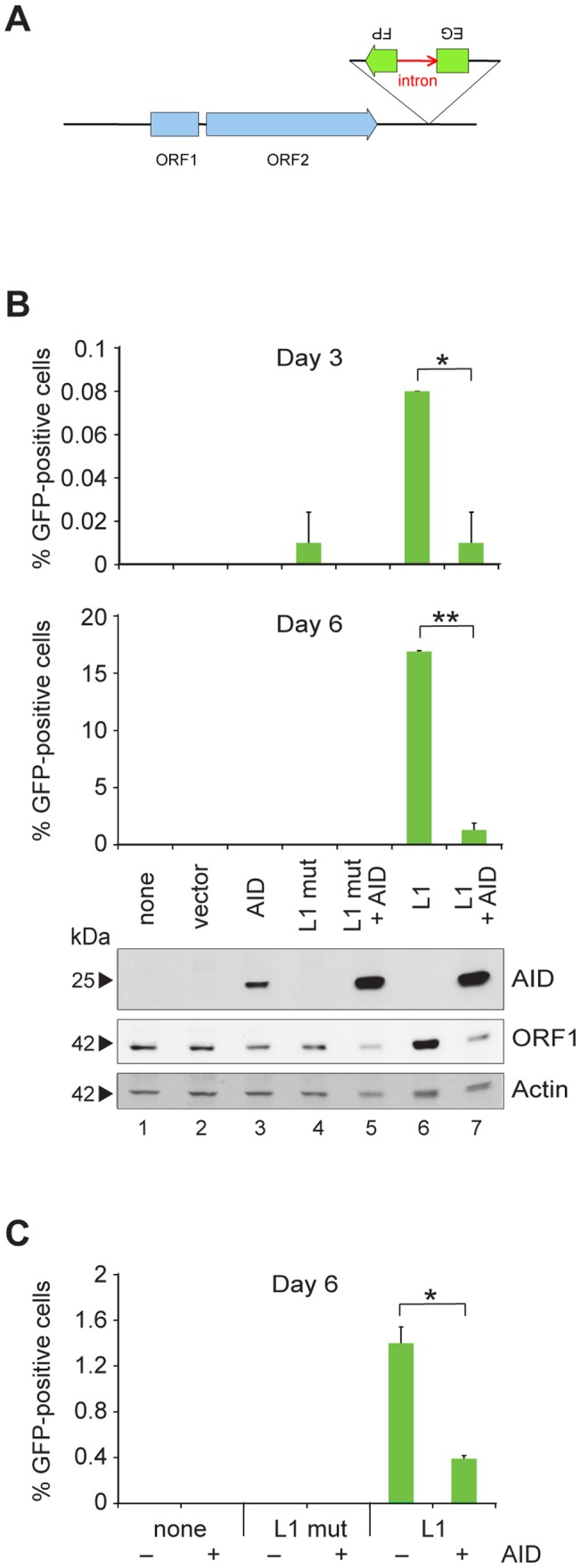
AID inhibits L1 retrotransposition. (**A**) L1 retrotransposition reporter construct. The plasmid contains the human L1_RP_ element with its proteins, ORF1 and ORF2. The construct also contains a puromycin resistance marker to select for transfected cells (not shown). The intron (in red) in the EGFP is in the same transcriptional orientation as ORF1 and ORF2 (indicated by the arrowhead of the ORF2 exon). When transcribed from the L1 promoter, the GFP gene is spliced, but it cannot be translated, as it is in the inverse orientation and does not encode functional protein. When transcribed from the EGFP promoter (opposite orientation; indicated by the arrowhead of the second EGFP exon), it cannot be spliced and thus not translated. Only when the L1 transcript is spliced, reverse-transcribed and inserted into the genome (a retrotransposition event) is GFP protein expressed. (**B**) Retrotransposition assay in HEK293 cells. Above, GFP-positive cells as determined by flow cytometry on days 3 and 6 after transfection of the reporter construct (L1) and/or AID. L1 mut is a reporter construct in which the ORF1 contains two missense mutations. Scales on the y-axis differ for day 3 and day 6. Values represent mean ± SD; n = 2 transfection samples. Student's t test; *p≤0.01 and **p≤0.001. Below, Western blots of lysates from cells 3 days after transfection, developed with anti-AID, anti-ORF1, or anti-actin antibody. The position of the molecular mass standard (in kDa) is indicated next to the blots. (**C**) Retrotransposition assay in WEHI-231 cells. GFP-positive cells as determined by flow cytometry on day 6 after transfection of the reporter constructs L1 and L1 mut. +, AID-positive WEHI-231 subclone; –, AID-negative WEHI-231 subclone. Values represent mean ± SD; n = 2 transfection samples. Student's t test; *p≤0.01.

## Materials and Methods

### Ethics statement

This study was approved by the UCSF Institutional Animal Care and Use Committee (IACUC). All experiments with mice were performed following the protocol AN083218-03 most recently re-approved by the UCSF IACUC in February 2012, as a yearly renewal. UCSF's animal house abides by all governmental regulations and standards, Animal Welfare Assurance Number A3400-01.

### L1 retrotransposition assay

The L1-GFP reporter construct L1 (kindly provided by Haig Kazazian, Jr., and described in [24]) was transfected into HEK293 cells (Lipofectamine; Invitrogen) and WEHI-231 cells (Neon, setting: 1350 V–30 ms –1 pulse; Invitrogen) in the presence or absence of AID. After 24 h the cells were put under antibiotic selection: HEK293 cells, 1 µg/ml puromycin for the L1 construct, and 600 µg/ml G418 for the AID construct; WEHI-231 cells, 0.5 µg/ml puromycin for the L1 construct. On days 3 and 6 after transfection the positively selected cells were analyzed by flow cytometry; the GFP-positive cells reflected L1 retrotransposition events. A reporter construct, L1 mut (also provided by Haig Kazazian, Jr., and described in [24]), with two missense mutations in the ORF1 (and thus nonfunctional) served as a negative control.

### AID induction in stably transfected HeLa cell clones

HeLa cell clones, stably transfected with a doxycycline-inducible mouse wild-type AID construct [25], were induced with 1 µg/ml doxycycline. After 24 h, AID-expressing cells were harvested for further experiments.

### AID and L1 ORF1 transfection of Phoenix cells

Ecotropic-package Phoenix cells (of HEK293 cell origin) were transiently transfected with 50 µl Lipofectamine (Invitrogen) and 12 µg plasmid DNA per 10 cm cell culture dish. The plasmid DNA was retroviral pCru based [26] and encoded: mouse wild-type AID protein with or without N-terminal Flag-tag; ORF1 protein of the human L1.3 element [L1.3 sequence, Genbank:L19088]; and, as a control, cells were transfected with the pCru plasmid encoding GFP with or without N-terminal Flag-tag. The cells were harvested 72 h after transfection and subjected to subsequent immunoprecipitation and Western blot experiments. The viral supernatant was harvested 48 and 72 h after transfection and used for retroviral infection of primary B cells.

### AID induction in primary B cells

Primary splenic B cells from BALB/c mice were cultured in standard medium and stimulated with 25 µg/ml LPS (Sigma) and 10 ng/ml IL-4 (R&D Systems). After 2–4 days, the activated B cells were subjected to retroviral infections or harvested. As controls, B cells from age- and sex-matched AID knockout BALB/c mice (kindly provided by Fred Alt; described in [2]) were cultured under the same conditions. All mouse protocols were approved by the Institutional Animal Care and Use Committee of the University of California, San Francisco.

### Retroviral infection of primary B cells

B cells that had been isolated from mouse spleens and stimulated with LPS and IL-4 for 2 days were spinoculated with viral supernatant for 1 h at 1,900×*g* at room temperature in the presence of 4 µg/ml polybrene. Forty-eight hours after infection, the cells were harvested and used in subsequent experiments.

### Gamma irradiation of primary B cells

B cells (1×10^7^) obtained from mouse spleens and stimulated with LPS and IL-4 for 4 days were subjected to 0, 1 or 50 Gy of gamma radiation. Twenty-four hours later, irradiated cells were harvested for Western blot analysis.

### Flow cytometry

For detection of GFP fluorescence, cells were washed and resuspended in FACS buffer (PBS, 2% FCS, 0.05% NaN_3_), followed by measurement on a FACSCalibur flow cytometer (BD Biosciences). Data were analyzed using CellQuestPro (BD Biosciences).

### Western blotting

Cell lysates, immunoprecipitates and FPLC fractions were analyzed by SDS-PAGE, followed by transfer to a nitrocellulose membrane. For protein detection, the following antibodies were used: anti-hA3G (Abcam, cat. #ab54257); anti-actin (Oncogene Research, cat. #CP-01); anti-AID (AIDA 94.16 [27]); anti-Flag (Sigma, cat. #F1804); anti-human ORF1 [28] (kindly provided by Gerald Schumann); and anti-mouse ORF1 [29], [30] (kindly provided by Sandra Martin).

### Quantitative real-time RT-PCR

Total mRNA was isolated using the RNeasy Kit (Qiagen). After digestion with DNase I (Ambion), the mRNA was reverse-transcribed into single-stranded cDNA using the SuperScript III First Strand Synthesis System for RT-PCR (Invitrogen), followed by quantitative PCR with the Applied Biosystems 7300 real-time PCR cycler. A gene-specific forward and reverse primer (Applied Biosystems) and a gene-specific FAM-labeled MGB probe (Applied Biosystems) were used. Mouse IAP, IAPfor TGCTAATTTTACCTTGGTGCAGTTA, IAPrev GTTTGCCAGTCAGCAGGAGTTA, IAP probe ACAGGCTCGCCGGCATGGC [31]; human L1, hL1for AAGCTACCAATGACTTTCTTCACAGAA, hL1rev CAGCTTTGTTCTTTTGGCTTAGGAT, hL1 probe, ATGCGGGCTCTTTTTTGGT; mouse L1, mL1for CTAGGATAGCAAAAAGTCTTCTCAAGGA, mL1rev GCAGTTTTTATCACAATTGCTCTGTAGT, mL1 probe, CCGCCAGAAGTTCTTT; MLV, MLVfor AAGCGGGTGGAAGACATCC, MLVrev AGCCCGCTCAAGAGGTTGT, MLV probe CCCCACCGTGCCCAACCCT [32]; human GAPDH (Applied Biosystems, cat. #Hs02758991_g1) and mouse 18S ribosomal RNA (Applied Biosystems, cat. #Mm03928990_g1) were used as endogenous controls. Quantitative real-time PCR data were analyzed according to the comparative *C*_T_ method.

### Protein and RNA immunoprecipitation

In some cases, cells were UV-cross-linked using the Stratalinker from Stratagene (UVC 254 nm; 60 mJ/cm^2^). Cells were lysed in the presence of RNase inhibitors (Promega) and incubated with 5 µg precipitating antibody (mouse monoclonal anti-AID antibody AIDA 94.16 or mouse monoclonal anti-Flag antibody [Sigma]), followed by incubation with protein G agarose (Invitrogen, cat. #15920-010). For protein IP, the precipitates were subjected to Western blot analysis; for RNA IP, the precipitates were digested with DNase I and the RNA was reverse-transcribed into cDNA using the SuperScript III First Strand Synthesis System for RT-PCR from Invitrogen, with oligo(dT) primers. RT-PCR was performed with the following primers: L1, L1for 5′ CCATGCTCATGGATTGG, L1rev 5′ ATTCTGTTCCATTGGTCTA [33]; germ line transcript γ1, GLTfor 5′ GGCCCTTCCAGATCTTTGAG, GLTrev 5′ GGATCCAGAGTTCCAGGTCACT; Ig κ-light chain, κfor 5′ GGCTGCAG(GC)TTCAGTGGCAGTGG(AG)TC(TA)GG(AG)AC, κrev 5′ GTGGTGGCGTCTCAGGACCTTTGT; Ig µ-heavy chain, µfor 5′ AGAGTCAGTCCTTCCCAAATGTCTTC, µrev 5′ TCCATGTGACATTTGTTTACAGCTCAGC; GAPDH, GAPDHfor 5′ TGAAGGTCGGTGTGAACGGATTTGGC, GAPDHrev 5′ CATGTAGGCCATGAGGTCCACCAC. After agarose gel electrophoresis, the resulting bands were cloned into a pCR2.1-TOPO sequencing vector using the TOPO TA cloning system (Invitrogen) and sequenced.

### Fast protein liquid chromatography

Cells were lysed in FPLC lysis buffer (50 mM HEPES, pH 7.4, 125 mM NaCl, 0.2% NP-40, 5 mM MgCl_2_, 1 mM phenylmethylsulfonyl fluoride, EDTA-free Complete Mini protease inhibitors); nuclei were removed and the remainder was passed over a Superose 6 HR 10/30 column (GE Healthcare) that separates molecules according to size using an AKTA purifier (GE Healthcare). FPLC running buffer: 50 mM HEPES, pH 7.4, 125 mM NaCl, 1 mM dithiothreitol, 10% glycerol. Protein fractions were collected and subjected to SDS-PAGE. In some cases, cell lysates were treated with 100 µg/ml RNase A (Roche) and RNase inhibitors (Promega) before fractionation.

### Statistical analysis

Statistical significance was determined using the Student's t test.

## Results

### AID inhibits L1 retrotransposition

AID has been reported to be as efficient as A3A in inhibiting L1 elements in HEK cells [23]. We set out to confirm these findings and to extend them to more physiological conditions in a B lymphocyte line, since B cells are the primary site of AID expression. To demonstrate that L1 retrotransposition is inhibited by AID, we used a construct containing a genetically marked L1 retroelement [24] (Figure 1A). Unlike endogenous retroviruses, this retroelement does not leave the cells but proliferates via retrotransposition. In the reporter construct, the marked element contains an enhanced GFP (EGFP) gene for green fluorescent protein in reverse orientation to the retroelement coding sequence; the GFP gene is disrupted by a forward-facing intron that precludes its expression from the construct. After transcription of the marked element into mRNA, splicing out of the intron, reverse transcription, and integration into the host cell genome, the genomic GFP gene becomes active (Figure 1A). In short, GFP can be expressed only if its transcript has undergone the intermediate cDNA step during an L1 retrotransposition event. Cells transfected with an L1 construct that is not transposition competent (L1 mut) served to assess the frequency of false-positive retrotranspositions. To confirm that AID protein is actually expressed in HEK cells transfected with AID, we performed AID-specific Western blot analyses (Figure 1B, lower panel, lanes 3, 5, 7).

We introduced the L1 reporter construct, with or without the AID gene, into HEK cells and selected for transfected cells, which we then analyzed by flow cytometry to measure the retrotransposition frequencies, indicated by GFP fluorescence. In these experiments, the false-positive retrotransposition frequency for each time point did not exceed 0.01% (L1 mut, Figure 1B and C). On day 3 after transfection, 0.08% of the cells containing the functional L1 construct were GFP positive; that is, in their genome there was a de novo L1 integration event. In the presence of AID, however, only 0.01% of cells were green, representing an 8-fold reduction in L1 integrations (p = 0.01; Figure 1B, upper graph). By day 6 after transfection, 17% of the cells contained retrotransposition events; in the presence of AID, this frequency was reduced to 1% (17-fold) (p = 0.0004; Figure 1B, lower graph). To exclude the possibility that the reduced GFP expression in cells with AID may have been caused by an AID activity on plasmid DNA rather than one specific for L1, we now used GFP expression plasmids instead of the L1 reporter constructs for the experiment. We found similar percentages of GFP-positive cells in the presence and absence of AID (see Figure S1). We thus confirmed the results of MacDuff et al. that AID restricts L1 retrotransposition in HEK cells [23].

We also performed the retrotransposition assay in endogenous AID-positive and AID-negative subclones of the mouse B-cell line WEHI-231 (Figure 1C). In these cells, AID reduced the retrotransposition by 3.5-fold (p = 0.005).

### AID decreases the steady-state level of L1 protein

On the Western blots of Figure 1B, the steady-state level of open reading frame 1 (ORF1) protein of L1 is reduced in the presence of AID (compare lanes 6 and 7). During the L1 replication cycle, L1 DNA is transcribed into mRNA, transported to the cytoplasm and translated into two proteins, ORF1 and ORF2; L1 RNA and its proteins interact by forming L1 ribonucleoprotein particles, with ORF1 as the RNA-binding component and ORF2 as the endonuclease and reverse transcriptase required for integration into the host genome [11]. Since ORF2 protein cannot be detected by Western blot analysis, owing to either inefficient translation or rapid elimination after translation [34], we focused on ORF1. The reduction of ORF1 in the presence of AID (Figure 1B, lanes 6 and 7) paralleled the reduction in numbers of GFP-positive cells reflecting retrotransposition events. However, we believe that most of the reduction in ORF1 protein level was not simply due to a reduction in retrotransposition, but to a more direct effect of AID on ORF1 protein; on day 3, only approximately 0.1% of the cells had a retrotransposition event, although all of them ought to contain the transfected reporter episome. The ORF1 level is, therefore, probably due to transcription and translation off the episome and not off the few new genomic integrations. In line with this, the steady-state level of endogenous ORF1 protein (Figure 1B, lane 4) was also reduced by AID (lane 5). It is unlikely that this reflects a (substantial) decrease in the number of endogenous retrotransposition events within 3 days. If the endogenous L1 retroelements were so active in HEK cells that the level of ORF1 protein would increase about 3-fold in 3 days (Figure 1B, compare lanes 5 and 4), then the genome would be quite unstable. We, therefore, assumed that AID may reduce the level of ORF1 protein, which is needed for retrotransposition [11], [35]; ORF1 not only binds to L1 mRNA, but is also thought to play a role in the recruitment of ORF2 to the L1 mRNA and their transport to the nucleus [35].

**Figure 2:**
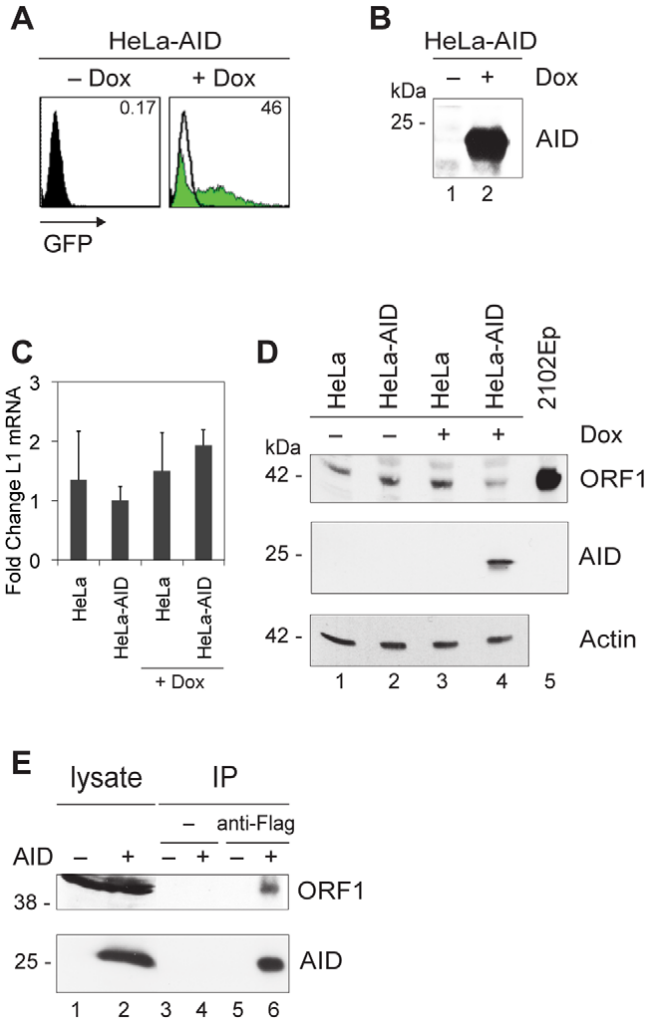
AID decreases the steady-state level of L1 protein. The doxycycline (Dox)-inducible construct encodes AID and GFP, expressed as two proteins; HeLa, untransfected HeLa cells; HeLa-AID, HeLa clone stably transfected with the inducible AID construct; – Dox, uninduced cells; + Dox, induced cells. (**A**) AID induction in HeLa cells: flow cytometry analysis. GFP expression (x-axis) as an indicator of AID expression. Numbers represent the percentage of GFP-positive cells; empty and black parts of the histogram are – Dox. (**B**) AID induction in HeLa cells: Western blot analysis. HeLa cell lysates were electrophoresed, Western blotted and developed with monoclonal anti-AID antibody. The position of the molecular mass standard (in kDa) is indicated next to the blot. (**C**) L1 mRNA levels in HeLa cells. After digestion with DNase, mRNA of HeLa cells was reverse-transcribed into cDNA using oligo(dT) primers, followed by L1-specific quantitative real-time PCR. PCR data were quantified according to the comparative *C*_T_ method. The amount of L1 target mRNA was normalized to the endogenous control GAPDH. Values represent mean ± SD; n = 3 independent experiments; y-axis, fold change in L1 mRNA expression (HeLa-AID set to 1.0). (**D**) ORF1 protein levels in HeLa cells. HeLa cell lysates were electrophoresed, Western blotted and developed with anti-ORF1, anti-AID or anti-actin antibody. 2102Ep, lysate from a human embryonal carcinoma cell line expressing high levels of ORF1. The position of the molecular mass standard (in kDa) is indicated next to the blots. (**E**) Western blot developed with ORF1 (above) and AID (below). ORF1 protein was co-precipitated with AID in lysates of Flag-AID (+)- and Flag-GFP (−)-transfected Phoenix cells (derived from HEK293 cells). Lysate, input used for IP; IP, immunoprecipitates; anti-Flag, immunoprecipitation with Flag-specific antibody; –, immunoprecipitation control without antibody. The position of the molecular mass standard (in kDa) is indicated next to the blots.

In the following experiments, we wanted to study the effect of AID on the steady-state level of endogenous L1 ORF1 protein product. We used inducible HeLa-AID cells, which contain a tetracycline-inducible AID-GFP construct [25]. After the tetracycline analog doxycycline is added for 24 h, AID and GFP genes are transcribed off a bidirectional promoter, and are expressed as two proteins. There was little leakiness of AID/GFP expression without doxycycline–at most, 0.17% cells were AID- or GFP-positive without the drug (Figure 2A). The induction with doxycycline resulted in 46% of the cells being AID/GFP-positive (Figure 2A); on Western blots developed with anti-AID antibody, the induced cells gave a strong AID band (Figure 2B), whereas the uninduced cells did not. The L1 mRNA of uninduced and induced cells, with or without AID, was about the same (Figure 2C). These cells, therefore, were suitable for our purpose: to follow endogenous ORF1 protein levels in the absence and presence of AID. On Western blots developed with an anti-ORF1 antibody, AID-negative (uninduced) HeLa cells gave an ORF1 band at 42 kDa (Figure 2D, lane 2), which was greatly reduced in AID-positive (induced) HeLa cells (Figure 2D, lane 4). Doxycycline itself did not affect the L1 protein (Figure 2D, lane 3 vs. lanes 1 and 2). We therefore concluded that AID reduces the L1 protein level. We also have some indication that AID interacts with L1 ORF1 protein, either directly or indirectly. We performed a conventional anti-Flag co-immunoprecipitation (co-IP) in HEK cells transfected with Flag-AID or Flag-GFP. The co-IP resulted in the pull-down of endogenous ORF1 protein with AID (Figure 2E, lane 6).

### Activated B lymphocytes express L1 mRNA, but not L1 protein

But how is the level of L1 ORF1 protein reduced in the presence of AID? There are several indications of AID binding to RNA. First, AID expressed in insect cells needs to be treated with RNase to become catalytically active on single-stranded DNA in vitro [36]. Second, the C terminus of AID binds to polyA-containing mRNA [37]. Third, APOBEC family members interact with RNA: A1 deaminates an RNA substrate, such that a premature stop codon is introduced. This results in a shorter apoB protein with a function in lipid metabolism different from that of the longer protein [38]–[41]. Because it was suggested that, similar to A1, AID may also mutate the RNA [42], a premature stop codon may be generated, which, in turn, would lead to nonsense-mediated RNA degradation. Even without introduction of a stop codon, inhibition of translation might result in increased degradation of mRNA and thus decreased steady-state protein levels. Although we did not find a significant difference in the presence or absence of AID in HeLa cells with quantitative real-time RT-PCR measurements of L1 mRNA (Figure 2C), we wanted to study this in more detail in activated B cells – the cells in which AID is mainly expressed.

**Figure 3:**
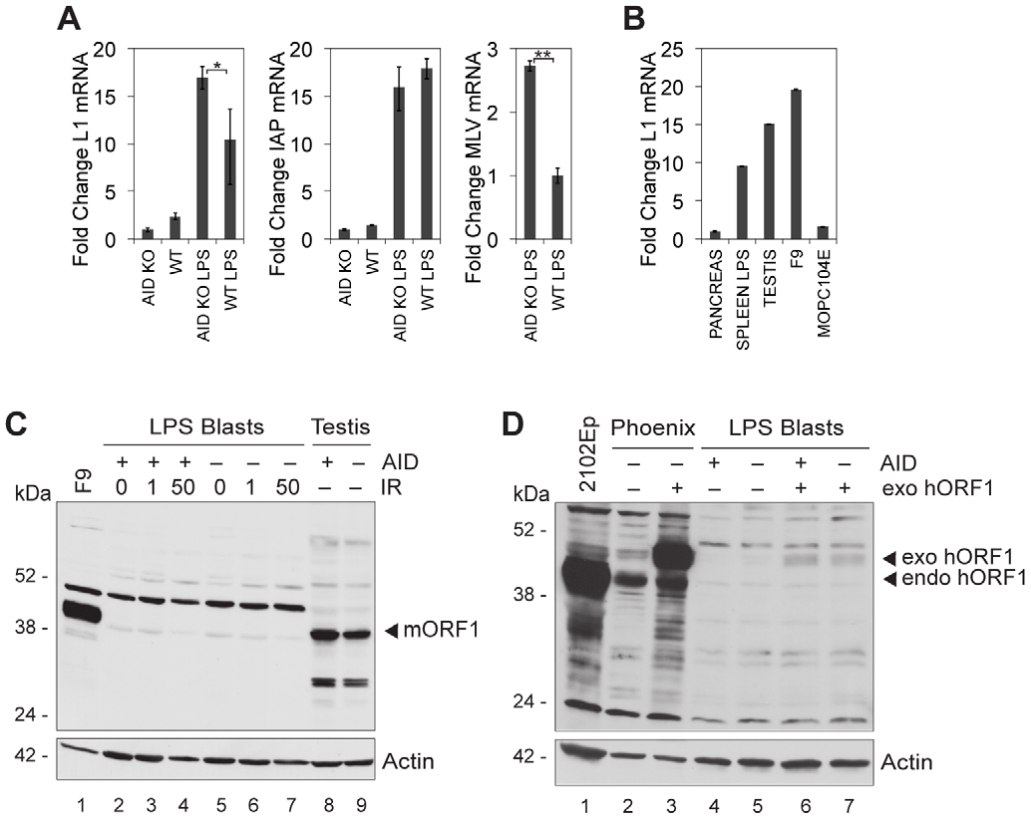
Activated B lymphocytes express L1 mRNA, but not L1 protein. (**A and B**) L1, IAP and MLV mRNA expression. After digestion with DNase, mRNA was reverse-transcribed into cDNA using oligo(dT) primers, followed by L1-, IAP- and MLV-specific quantitative real-time PCR. PCR data were quantified according to the comparative *C*_T_ method. The amount of target mRNA was normalized to the endogenous control 18S ribosomal RNA. Values represent mean ± SD; y-axis, fold change in mRNA expression. (A) LPS- plus IL-4-stimulated (LPS) and unstimulated B lymphocytes from the spleens of AID-deficient (AID KO; n = 12 mice) and wild-type (WT; n = 12 mice) BALB/c mice. Student's t test, *p≤0.05 and **p≤0.001. (B) Tissues (pancreas, testis) and activated B cells (spleen LPS) from a wild-type BALB/c mouse and mouse cell lines F9 and MOPC104E; F9, mouse embryonal carcinoma cell line expressing high levels of L1 mRNA; spleen LPS, LPS- plus IL-4-stimulated B lymphocytes, as in panel A; n = 2 independent experiments. (**C and D**) ORF1 protein expression. Cell lysates were electrophoresed, Western blotted and developed with anti-ORF1 or anti-actin antibody. The position of the molecular mass standard (in kDa) is indicated to the left of the blots; the positions of the exogenous (exo) and endogenous (endo) human (hORF1) and mouse (mORF1) ORF1 bands are indicated to the right of the blots; LPS blasts, lysates of LPS- plus IL-4-activated B lymphocytes from BALB/c mice; + AID and – AID, AID-sufficient and AID-deficient, respectively. (C) anti-mORF1 staining; testis, whole testis lysates; IR, cells gamma irradiated 24 h before lysis with 0, 1 or 50 Gy. (D) anti-hORF1 staining; 2102Ep, lysate from a human embryonal carcinoma cell line expressing high levels of ORF1; Phoenix and LPS blasts, lysates of cells transfected (Phoenix) or transduced (LPS Blasts) with a retroviral human ORF1 (+ exo hORF1) or a GFP-only construct (− exo hORF1).

When we stimulated spleen cells with lipopolysaccharide (LPS), L1 mRNA levels apparently increased about 4-fold in the presence of AID and about 17-fold in its absence (Figure 3A). However, while only B cells start to proliferate and thus yield a homogeneous population of blasts after 3 days in culture, the spleen contained only 25% uninduced B cells; the rest of the cells may have contained various amounts of L1 mRNA. This precludes firm conclusions about a change in L1 mRNA levels upon B-cell stimulation. However, we consider the 1.6-fold increase in L1 mRNA level in AID-deficient compared to AID-sufficient LPS blasts to be significant, even though we cannot correlate it with protein expression, which is absent in blasts (see below). AID made no difference in the levels of mRNA encoding intracisternal A particles (IAP), but it reduced levels of murine leukemia virus (MLV) mRNA by 2.7-fold (Figure 3A).

The level of L1 mRNA in LPS blasts does not differ much from that in testis or in F9 teratocarcinoma cells (Figure 3B), both of which express high levels of ORF1 protein [29], [30]. We thus were surprised that no ORF1 protein was expressed in the blasts, as assessed by Western blot analysis (Figure 3C, lanes 2 and 5). In contrast, the F9 and testis cells yielded a strong ORF1 band at 41 kDa (Figure 3C). However, a weaker band at 50 kDa, of unknown identity, was present in both F9 cells and LPS blasts, but not in testis cells. We then tried to induce retroelements by gamma irradiation, which increases L1 mRNA levels and L1 retrotransposition [43], [44]. However, the irradiated blasts still did not give any ORF1 protein bands (Figure 3C, lanes 3, 4, 6, and 7). Although we tentatively concluded that no ORF1 protein is expressed in blasts, we cannot exclude the possibility that the antibody detects only a subset of the many different mouse ORF1s. We therefore introduced human ORF1 into LPS blasts (Figure 3D; for infection efficiencies, see Figure S2) and analyzed protein expression by means of Western blots developed with antibody to human ORF1; the human embryonal carcinoma cell line 2102Ep, which expresses high levels of ORF1, provided the marker for the 41-kDa protein (Figure 3D, lane 1). The human Phoenix line produces some endogenous ORF1 protein (lane 2), which differs in molecular mass from the exogenous one, at 46 kDa. Only faint bands at the relevant position were seen in the LPS blasts transduced with the human ORF1 construct (Figure 3D, lanes 6 and 7; more samples, see Figure S2). Therefore, we conclude that ORF1 protein is not expressed at a significant level in LPS blasts, even in the absence of AID. The possibility remains that activators other than LPS or gamma irradiation would induce protein production.

### AID forms high-molecular-mass complexes in the cytoplasm

**Figure 4:**
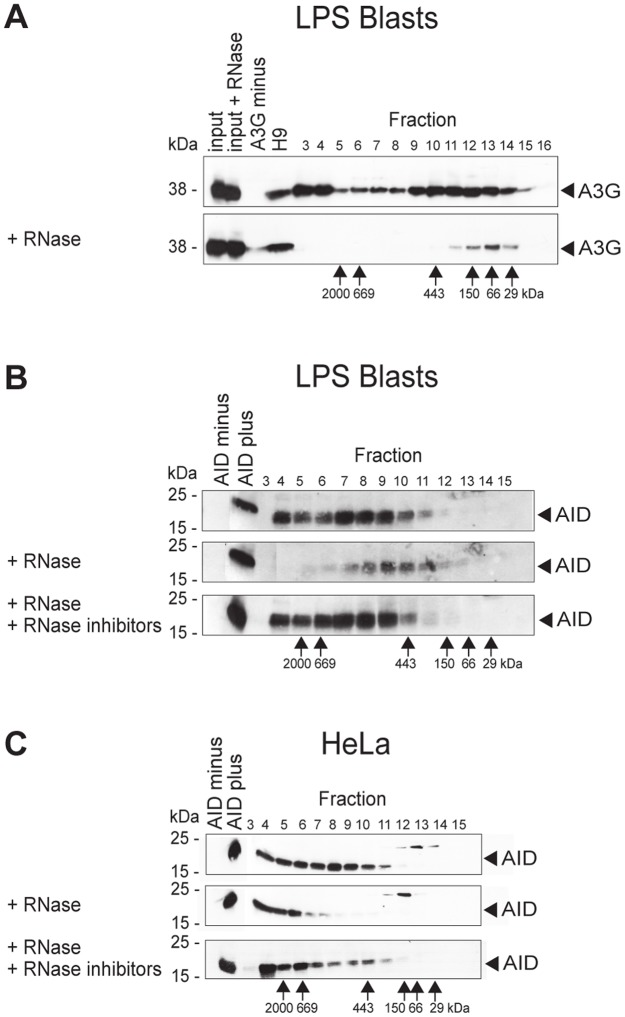
AID forms high-molecular-mass complexes in the cytoplasm. Fractionation according to size by gel filtration of A3G (A) and AID (B and C), followed by Western blot analysis. Numbers above panels, fractions of separation by FPLC; numbers to the left of panels, molecular mass standards (in kDa) of the SDS gel run; numbers below panels, molecular mass standards (in kDa) of the fractions of the FPLC run. (**A**) Western blots of fractions of FPLC eluates were developed with polyclonal antibody to A3G. Input, untreated lysate of A3G-positive cells; input + RNase, RNase A-treated lysate of A3G-positive cells; A3G minus, lysate of A3G-negative cells; H9, A3G-positive cell line; fractions 3–16, fractionated lysates of LPS- plus IL-4-activated B lymphocytes from human A3 transgenic mice. Upper panel, untreated cell lysate (devoid of nuclei); lower panel, treated with RNase A before fractionation on FPLC. (**B and C**) Western blots of fractions of FPLC eluates developed with monoclonal antibody to AID. AID minus, lysate of AID-negative HeLa cells; AID plus, lysate of AID-positive HeLa cells; fractions 3–15, fractionated lysates of LPS- plus IL-4-activated B lymphocytes from AID-sufficient mice (B) and of AID-positive HeLa cells (C). Top panel, untreated cell lysate (devoid of nuclei); middle panel, treated with RNase A before fractionation on FPLC; bottom panel, treated with RNase A and RNase inhibitors before fractionation. Concomitant with RNase A treatment, proteins were somewhat digested for unknown reasons.

Investigating the mechanism of L1 inhibition by AID in more detail, we focused on cytoplasmic AID, which by far outweighs the enzyme present in the nucleus [45], [46]. The preponderance of cytoplasmic AID is an odd finding, considering that AID's known function as a DNA mutator [36], [47], [48] takes place in the nucleus. It has been reasoned that AID is retained in the cytoplasm so as not to damage the DNA; however, AID is synthesized only in activated B lymphocytes, in which AID is supposed to be enzymatically active. Here we took a cue from the characteristics of A3G, which restricts endogenous retroelements by sequestering their RNA into large complexes in the cytoplasm, thus preventing new genomic integrations [20], [21]. We thus set out to examine whether AID also forms cytoplasmic complexes. We first repeated the gel filtration experiments that demonstrated A3G complex formation [21], [49]. We lysed the cells, removed the nuclei and passed the remainder over a fast protein liquid chromatography (FPLC) column that separates molecules according to size, under conditions as published [49]. By doing so, we confirmed that A3G is present as large complexes in the cytoplasm of activated B cells from human A3 transgenic mice (Figure 4A, upper panel). Since these complexes contain RNA, they fell apart upon treatment with RNase A (Figure 4A, lower panel).

In analogous experiments with activated B cells from AID-sufficient wild-type mice, we also found AID in large complexes (Figure 4B, upper panel). Because most AID is located in the cytoplasm, and we had depleted the cell lysate of the nuclei, the complexes described here ought to be mainly cytoplasmic. Most AID was part of a complex of medium molecular mass (>400 kDa) or high molecular mass (>2 MDa). When treated with RNase before separation on the column, the large AID complexes in the cytoplasm dissociated (Figure 4B, middle panel), analogous to the A3G complexes in Figure 4A, albeit not yielding 24-kDa monomeric AID. With RNase plus RNase inhibitor, the AID complexes were stable (Figure 4B, lower panel). To see whether ectopic AID in non-B-cell lines from our previous experiments (Figures 1, 2) also forms complexes, we performed FPLC fractionations with AID-positive HeLa cells. Again, we found AID in large complexes (Figure 4C, upper panel). When treated with RNase before separation on the column, however, the large AID complexes in HeLa cells did not dissociate (Figure 4C, middle panel) as they did in primary blasts (Figure 4B). Instead, the smaller complexes were absent, whereas the larger ones seemed unaffected (Figure 4C, middle panel). With RNase plus RNase inhibitor, the AID complexes were again stable (Figure 4C, lower panel). Although we do not know the reason for the difference in dissociation behavior of the AID complexes between HeLa cells and activated B lymphocytes, we concluded that AID, like A3G, forms cytoplasmic complexes that contain RNA.

### AID binds to L1 mRNA

**Figure 5:**
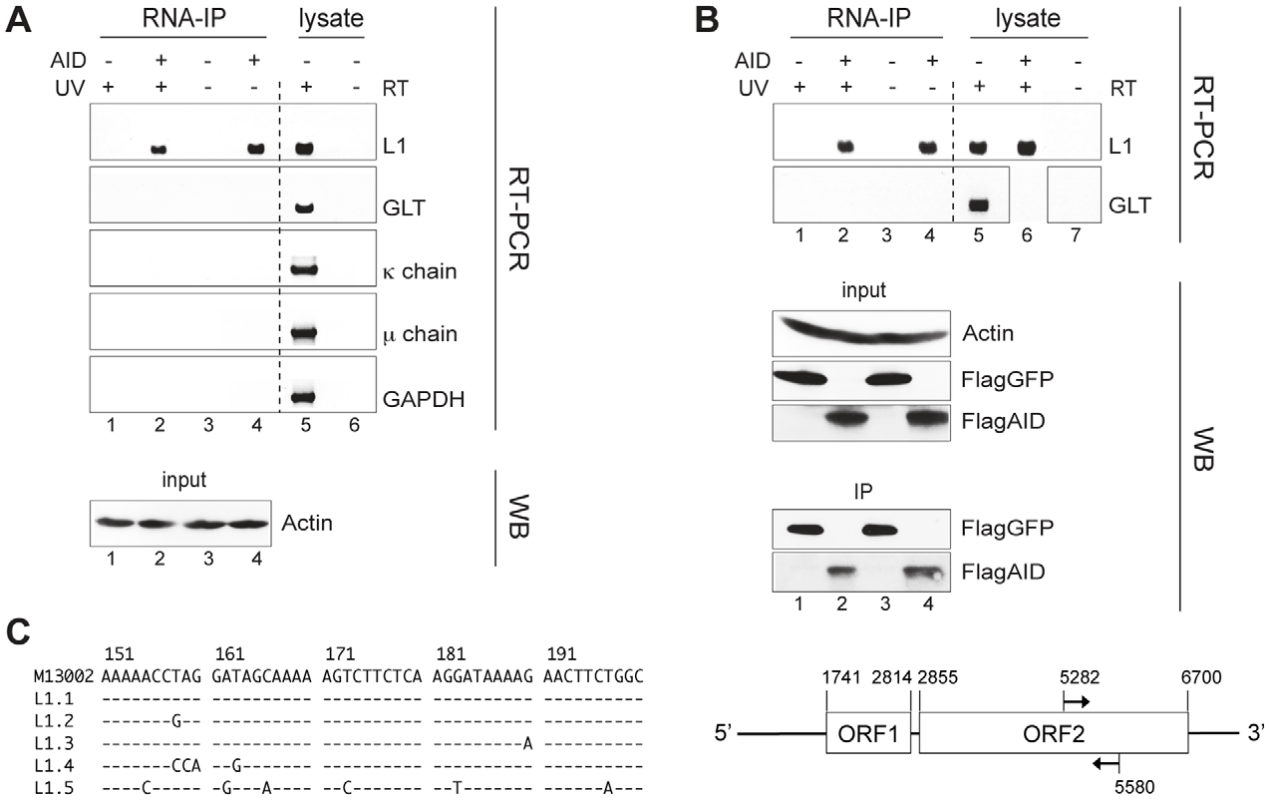
AID binds to L1 mRNA. (**A and B**) Agarose gels of products from RT-PCR on RNA-immunoprecipitates (RNA-IP) showing RNA binding for endogenous (A) and exogenous (B) AID from murine B lymphoblasts. Proteins and nucleic acids contained in 1×10^8^ LPS- plus IL-4-stimulated AID-deficient (AID −) and wild-type (AID +) B cells (A) or 7.5×10^7^ LPS- plus IL-4-stimulated AID-deficient B cells, transduced with Flag-GFP (AID −) or Flag-AID (AID +) (B), were cross-linked via treatment with UV (UV +), or not treated (UV−); the cells were then lysed, followed by immunoprecipitation with monoclonal anti-AID (A) or monoclonal anti-Flag (B) antibody. After DNase digest, RT-PCR using oligo(dT) primers, followed by L1-, germ line transcript (GLT)-, Ig κ-light chain (κ chain)-, Ig µ-heavy chain (µ chain)- or GAPDH-specific primers, was performed on the immunoprecipitates (“RT-PCR” panel, “RNA-IP”). To monitor the amount of AID in lysate and immunoprecipitates, we analyzed aliquots of lysates (WB panel, “input”) and immunoprecipitates (WB panel, “IP”), both equivalent to 5×10^6^ cells, and electrophoresed, Western blotted and developed them with an anti-actin, anti-Flag or anti-AID antibody. Lanes 1–4, RNA-IP samples; lanes 5–7, cDNA synthesis and PCR controls, i.e., total RNA of LPS- plus IL-4-stimulated AID-deficient and wild-type B cells with (+ RT) or without (− RT) reverse transcriptase. (**C**) Left: identity of L1 elements in the immunoprecipitates confirmed by cDNA sequencing. Sequences (nt 151–200) of the 300-bp L1 ORF2 fragments that were amplified from RNA-IP shown in panel A. M13002, L1 reference sequence of BALB/c strain origin; L1.1 and L1.2, sequences obtained from RNA-IP of lane 2 in panel A; L1.3 to L1.5, sequences obtained from lane 4 in panel A. Right: schematic of a full-length L1 element. Arrows indicate the position of the primers used to amplify the 300-bp L1 ORF2 fragment from RNA-IP shown in panel A and B. The numbers represent the nucleotide positions according to the L1Md-A2 sequence [L1 sequence, Genbank:M13002] [68].

We aimed to identify the RNA present in the large cytoplasmic AID complexes, in relation to AID's function in restricting retroelements. Since our data strongly indicated an inhibitory function of AID on L1 elements, we wanted to find out whether AID binds to L1 RNA. To isolate the RNA in the AID complexes, we performed RNA immunoprecipitation (RNA-IP) assays in activated mouse B lymphocytes stimulated with LPS and interleukin (IL)-4. In these immunoprecipitations, we pulled down RNA bound to precipitated AID (Figure 5A) or Flag-tagged AID/GFP (Figure 5B). We then digested the precipitate with DNase, reverse-transcribed the RNA into cDNA, and amplified the obtained cDNA with specific primers. Primary B lymphoblasts from AID-sufficient and -deficient mice both expressed high levels of L1 RNA (Figure 5A, lane 5; Figure 5B, lanes 5 and 6). However, we detected L1 sequences only in the AID immunoprecipitates of AID-sufficient cells (Figure 5A and B, lanes 2 and 4), not in the immunoprecipitates of AID-deficient cells (Figure 5A and B, lanes 1 and 3); no L1 product was amplified from irrelevant immunoprecipitates (i.e., Flag-GFP precipitates: Figure 5B, lanes 1 and 3). UV irradiation of the cells before lysis, as a means to potentially stabilize any AID – RNA interaction via cross-linking, apparently did not affect the amount of RT-PCR product (Figure 5A and B, lanes 2 and 4).

To test whether any abundantly expressed RNA could be amplified from AID immunoprecipitates without a specific interaction with AID, we did a series of additional RT-PCRs. First, we used germ line transcript (GLT)-specific primers. GLT is an RNA transcribed from a promoter 5′ to the constant region genes of the Ig heavy chain to which the B cell subsequently will switch. Because B cells stimulated with LPS and IL-4 predominantly switch to IgG1 production [50], [51], we focused on γ1 GLT. Although the cells do synthesize it in good amounts (Figure 5A and B, lanes 5), we did not find any γ1 GLT in the AID immunoprecipitates (Figure 5A and B, lanes 2 and 4). We obtained similar results with Ig κ-light chain-, Ig µ-heavy chain- and glyceraldehyde-3-phosphate dehydrogenase (GAPDH)-specific primers (Figure 5A). The L1 sequences amplified from endogenous and exogenous AID immunoprecipitates (Figure 5A and B) were confirmed by sequencing to be derived from different L1 elements (Figure 5C).

## Discussion

AID is known for its function in adaptive immunity by mutating the Ig locus in activated B lymphocytes and, thereby, generating the secondary antibody repertoire. A study published in 2006 added to this view by proposing that AID also responds to infection by Abelson virus [22]. AID would introduce mutations into the host genome, and the resulting genotoxic stress would lead to apoptosis of the infected cells [52]. Another publication also suggested a role for AID in innate immunity, demonstrating that ectopic AID inhibits retrotransposition of L1 elements in non-B-cell lines [23]. In the current study we confirmed that AID indeed inhibits L1 retrotransposition, in non-B- and B-cell lines. We also found that AID reduces ORF1 protein of L1, perhaps by binding to it.

Although AID expression has also been reported in germ cells and embryonic stem cells [6], [23], [53], [54], we did not find any AID protein in these cell types in the mouse (see Figure S3) and therefore focused on B lymphocytes. However, the retrotransposition assay in activated B cells poses technical difficulties – among them, finding transduction vectors that hold the large retrotransposition transporter constructs, and the short life span of primary cells in culture. We also did not identify conditions under which L1 ORF1 protein is expressed in activated B cells, despite mRNA levels similar to those in cells with strong ORF1 expression, such as testis cells. This may mean that, in the absence of AID, other restriction factors control L1. However, in cell lines and primary B-cell blasts, we established that AID forms large cytoplasmic complexes with L1 mRNA, but not with other mRNAs that are expressed at high levels.

But what is the mechanism of the inhibition of L1 retrotransposition by AID? AID binds to L1 mRNA and protein in the cytoplasm, thus preventing them from entering the nucleus and integrating their cDNA into genomic DNA. For suppression of Alu retrotransposition by A3G, the catalytic activity is dispensable [21], and AID may act similarly. AID may inhibit translation of ORF1, and/or the ORF1 protein may be targeted for degradation. While this manuscript was in preparation, Häsler et al. reported the existence of AID complexes in the cytoplasm [55] in agreement with our data. They showed that translation elongation factor 1alpha (eEF1A) binds to AID in these complexes. In several independent AID yeast two-hybrid screens with human or mouse AID as bait, we also found that eEF1A was the predominant hit; furthermore, eEF1A co-localized with the large and medium AID complexes from our FPLC fractionations (data not shown). Thus, eEF1A may function as a cytosolic retention factor for AID [55]. However, by binding to eEF1A, AID may also inhibit translation of L1 ORF1. AID may also recruit L1 RNA and protein into cytoplasmic complexes in order to degrade ORF1 protein; in addition to protein synthesis, eEF1A is believed to function in co-translational protein degradation [56].

As a DNA mutator, AID mediates switch recombination and hypermutation at the Ig loci. This activity can easily account for the hyper-IgM syndrome and inability to generate high-affinity antibody seen in AID-deficient patients [57]–[59]. But these patients also suffer from lymphadenopathies and autoimmune or inflammatory disorders (including diabetes mellitus, polyarthritis, autoimmune hepatitis, hemolytic anemia, immune thrombocytopenia, Crohn's disease and chronic uveitis) [58], [59] – seemingly disparate conditions that are at odds with the known function of AID. Also, AID knockout mice develop lymphoid hyperplasia and autoimmunity [60]–[62]. The lymphoid hyperplasia in AID-deficient humans [59], [63]–[65] and mice [61], [62] has been interpreted as being due to a lack of class-switched IgA, which would lead to an expansion of anaerobic commensal bacteria in the small intestine [61]. However, autoimmune disease is thought to be caused by high-affinity, not low-affinity, antibodies. Recent publications suggest that failure to inhibit retroelement activity can lead to autoimmune disease [16], [19], [66], [67]. We suggest that AID may protect B cells from the adverse effects of retroelements – intracellular accumulation of retroelements causing autoimmune disease, and insertional mutagenesis causing lymphoproliferative disease.

We found that AID is not only a DNA mutator that generates the secondary antibody repertoire, but is also a potent restriction factor for L1 retroelements. The restriction may be mediated by the cytoplasmic high-molecular-mass complexes that AID forms with L1 mRNA in activated B lymphocytes. But because AID-deficient activated B lymphocytes do not express ORF1 protein, we suggest that ORF1 protein expression is inhibited by additional restriction factors. The greater increase in MLV compared to L1 mRNA in AID-deficient activated B lymphocytes may indicate less strict surveillance of retrovirus.

### Supplementary Data

Supplementary Data are available at NAR online: Figures S1, S2, S3.

## Supporting Information

Figure S1 **AID activity on plasmid DNA.** GFP expression as determined by flow cytometry on day 6 after transfection of HEK293 cells with a GFP-encoding plasmid (GFP), in addition to an AID-encoding plasmid (AID) or empty vector (vector). Numbers in dot plots represent the percentage of GFP-positive cells; x-axis, GFP expression.
(TIF)

Figure S2 **Infection of activated B lymphocytes with a human ORF1 construct.** LPS- plus IL-4-stimulated AID-deficient (denoted AID KO on top of the flow cytometry plots of file 1A, and – AID on top of the Western blots of file 1B, respectively) and wild-type (WT and + AID, respectively) B cells from BALB/c mice were infected with a retroviral construct encoding human ORF1 (hORF1) and GFP, expressed as two proteins. (**A**) Infection efficiencies. GFP expression (x-axis) as an indicator of ORF1 expression. Numbers in dot plots represent the percentage of GFP-positive cells as determined by flow cytometry (mean WT-hORF1: 7.5%; mean AID KO-hORF1: 7%); #1–#6, independent infection samples; in Figure 3D, sample #2 is shown. (**B**) ORF1 protein levels. Cell lysates were electrophoresed, Western blotted and developed with anti-hORF1, anti-AID or anti-actin antibody. The position of the molecular mass standard (in kDa) is indicated to the left of the blots; the positions of the exogenous (exo) and endogenous (endo) human (hORF1) ORF1 bands are indicated to the right of the blots; numbers above the blots indicate the infection sample (1–6); 2102Ep, lysate from a human embryonal carcinoma cell line expressing high levels of ORF1; + exo hORF1, transduced with a retroviral human ORF1 construct; – exo hORF1, transduced with a retroviral GFP-only construct.
(TIF)

Figure S3 **AID protein expression in various cell types.** Cell lysates were electrophoresed, Western blotted and developed with anti-ORF1, anti-AID or anti-actin antibody. The position of the molecular mass standard (in kDa) is indicated to the left of the blots; lanes 1 and 2 are on another membrane than lanes 3–8; ES cells, lysates from mouse embryonic stem cells (left lane: E14; right lane: C57BL/6); testis, whole testis lysates from C57BL/6 mice; ovary, whole ovary lysates from C57BL/6 mice; LPS blasts, lysates of LPS- plus IL-4-activated B lymphocytes from BALB/c mice; + AID and – AID, AID-sufficient and AID-deficient mice, respectively.
(TIF)
