# Supplementary figures and images for: LINE-1 Retroelements Complexed and Inhibited by Activation Induced Cytidine Deaminase
Source: PLoS One. 2012 Nov 2;7(11):e49358. doi: 10.1371/journal.pone.0049358 (PMC3487726; doi:10.1371/journal.pone.0049358)

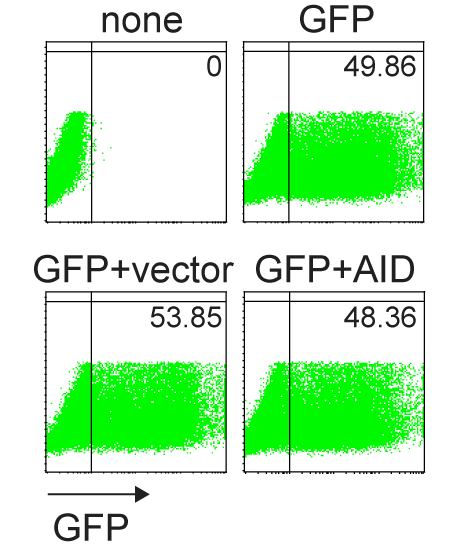

Supplement: Figure S1 — AID activity on plasmid DNA. GFP expression as determined by flow cytometry on day 6 after transfection of HEK293 cells with a GFP-encoding plasmid (GFP), in addition to an AID-encoding plasmid (AID) or empty vector (vector). Numbers in dot plots represent the percentage of GFP-positive cells; x-axis, GFP expression. (TIF) [file pone.0049358.s001.tif]

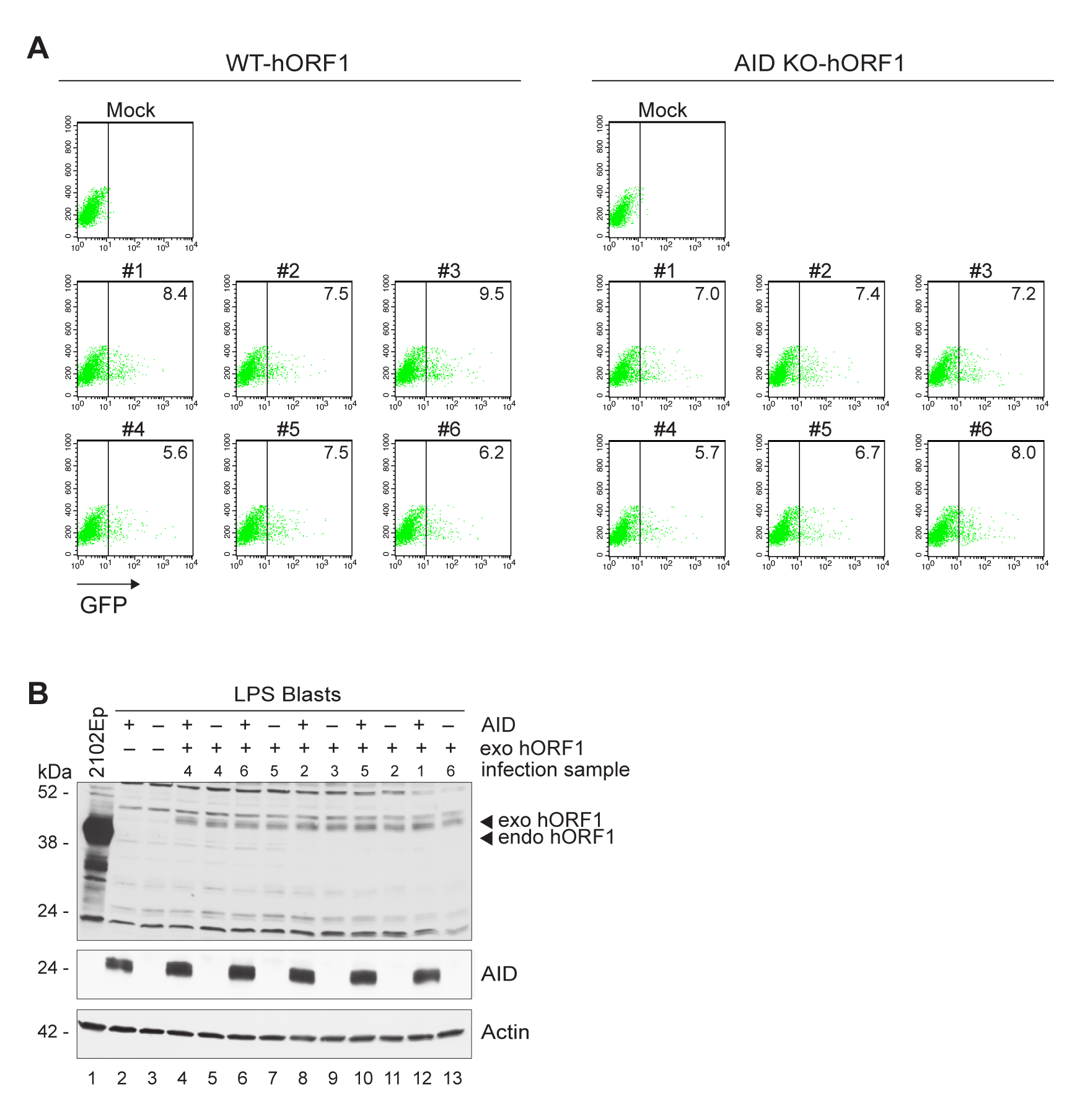

Supplement: Figure S2 — Infection of activated B lymphocytes with a human ORF1 construct. LPS- plus IL-4-stimulated AID-deficient (denoted AID KO on top of the flow cytometry plots of file 1A, and – AID on top of the Western blots of file 1B, respectively) and wild-type (WT and + AID, respectively) B cells from BALB/c mice were infected with a retroviral construct encoding human ORF1 (hORF1) and GFP, expressed as two proteins. (A) Infection efficiencies. GFP expression (x-axis) as an indicator of ORF1 expression. Numbers in dot plots represent the percentage of GFP-positive cells as determined by flow cytometry (mean WT-hORF1: 7.5%; mean AID KO-hORF1: 7%); #1–#6, independent infection samples; in Figure 3D, sample #2 is shown. (B) ORF1 protein levels. Cell lysates were electrophoresed, Western blotted and developed with anti-hORF1, anti-AID or anti-actin antibody. The position of the molecular mass standard (in kDa) is indicated to the left of the blots; the positions of the exogenous (exo) and endogenous (endo) human (hORF1) ORF1 bands are indicated to the right of the blots; numbers above the blots indicate the infection sample (1–6); 2102Ep, lysate from a human embryonal carcinoma cell line expressing high levels of ORF1; + exo hORF1, transduced with a retroviral human ORF1 construct; – exo hORF1, transduced with a retroviral GFP-only construct. (TIF) [file pone.0049358.s002.tif]

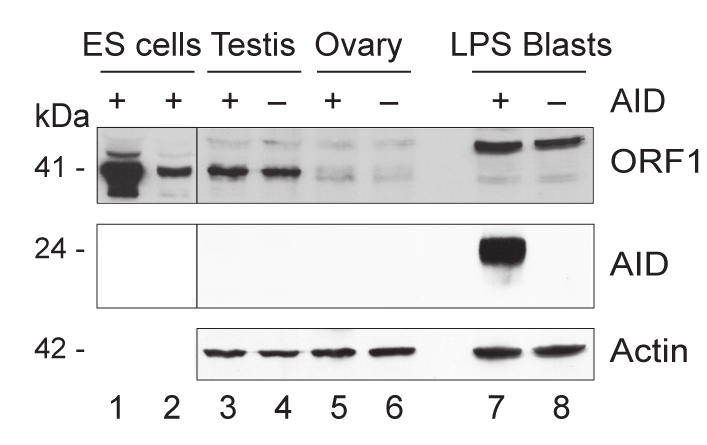

Supplement: Figure S3 — AID protein expression in various cell types. Cell lysates were electrophoresed, Western blotted and developed with anti-ORF1, anti-AID or anti-actin antibody. The position of the molecular mass standard (in kDa) is indicated to the left of the blots; lanes 1 and 2 are on another membrane than lanes 3–8; ES cells, lysates from mouse embryonic stem cells (left lane: E14; right lane: C57BL/6); testis, whole testis lysates from C57BL/6 mice; ovary, whole ovary lysates from C57BL/6 mice; LPS blasts, lysates of LPS- plus IL-4-activated B lymphocytes from BALB/c mice; + AID and – AID, AID-sufficient and AID-deficient mice, respectively. (TIF) [file pone.0049358.s003.tif]
